# *In Vitro* Endothelial Cell Proliferation Assay Reveals Distinct Levels of Proangiogenic Cytokines Characterizing Sera of Healthy Subjects and of Patients with Heart Failure

**DOI:** 10.1155/2014/257081

**Published:** 2014-03-23

**Authors:** Rebecca Voltan, Giorgio Zauli, Paola Rizzo, Alessandro Fucili, Micaela Pannella, Roberto Marci, Veronica Tisato, Roberto Ferrari, Paola Secchiero

**Affiliations:** ^1^Department of Morphology, Surgery and Experimental Medicine and LTTA Centre, University of Ferrara, 44121 Ferrara, Italy; ^2^Institute for Maternal and Child Health, IRCCS “Burlo Garofolo”, 34137 Trieste, Italy; ^3^Department of Medical Sciences, Cardiovascular Section, University Hospital, Arcispedale Sant'Anna and LTTA Centre, University of Ferrara, 44121 Ferrara, Italy; ^4^Maria Cecilia Hospital, GVM Care & Research, ES Health Science Foundation, Cotignola, 48010 Ravenna, Italy

## Abstract

Although myocardial angiogenesis is thought to play an important role in heart failure (HF), the involvement of circulating proinflammatory and proangiogenic cytokines in the pathogenesis and/or prognosis of HF has not been deeply investigated. By using a highly standardized proliferation assay with human endothelial cells, we first demonstrated that sera from older (mean age 52 ± 7.6 years; *n* = 46) healthy donors promoted endothelial cell proliferation to a significantly higher extent compared to sera obtained from younger healthy donors (mean age 29 ± 8.6 years; *n* = 20). The promotion of endothelial cell proliferation was accompanied by high serum levels of several proangiogenic cytokines. When we assessed endothelial cell proliferation in response to HF patients' sera, we observed that a subset of sera (*n* = 11) promoted cell proliferation to a significantly lesser extent compared to the majority of sera (*n* = 18). Also, in this case, the difference between the patient groups in the ability to induce endothelial cell proliferation correlated to significant (*P* < 0.05) differences in serum proangiogenic cytokine levels. Unexpectedly, HF patients associated to the highest endothelial proliferation index showed the worst prognosis as evaluated in terms of subsequent cardiovascular events in the follow-up, suggesting that high levels of circulating proangiogenic cytokines might be related to a worse prognosis.

## 1. Introduction

Heart failure (HF) is the final consequence of different heart diseases and it represents a prominent cause of morbidity and mortality worldwide [1]. At the cellular level, the occurrence of hypertrophy of cardiac muscle cells represents a common feature of failing myocardium [2, 3] and is considered as an adaptive response to increased external load in the presence of pathological situations such as hypertension or myocardial infarction. However, sustained overload eventually leads to contractile dysfunction and HF through incompletely understood mechanisms [4–6]. Dysfunctional vascular regulation is an important component of the pathophysiology of HF, and reduced levels of vascular endothelial growth factor (VEGF) have been observed in myocardium models of advanced HF [7]. Angiogenesis was enhanced during the acute phase of adaptive cardiac growth but reduced as hearts underwent pathological remodelling and it has been demonstrated that inhibition of VEGF signalling at the myocardium level leads to the transition from compensatory hypertrophy to cardiac failure [8], since both heart size and cardiac function are angiogenesis-dependent. A large number of preclinical studies have raised hope that increasing the expression of VEGF and/or other proangiogenic cytokines at the myocardial level show beneficial effects especially in animal models of postmyocardial infarction [7, 8]. Nevertheless, the clinical trials based on the proangiogenesis hypothesis have failed to provide conclusive results on the therapeutic benefits of clinical approaches aimed at improving myocardial angiogenesis especially in the early phases after myocardial infarction [7].

It should also be noticed that although most experimental studies point to a positive role of angiogenic cytokines at the cardiac level, few studies have addressed the potential role of circulating proinflammatory and proangiogenic cytokines in patients with HF. Since human serum contains a myriad of cytokines, a major limitation to the study of the proangiogenic capability of human serum is also due by the fact that most* in vitro* and* in vivo* angiogenic tests are complex and not easy to be reproduced [9, 10].

On these bases, in the present study, we have adopted a simple and reproducible* in vitro* endothelial cell proliferation assay in order to investigate the proangiogenic effects of human sera obtained from both healthy individuals and from a limited group of HF patients. The differential capability to promote* in vitro* endothelial cell proliferation was correlated with the presence and level of a variety of cytokines, analysed with the multiplex technology, and, for the HF patients, with relevant clinical parameters, such as NTpro-BNP levels and occurrence of cardiovascular events in the follow-up.

## 2. Methods 

### 2.1. Characteristics of the Healthy Subjects and Recruitment of HF Patients

The healthy group was represented by 66 subjects (age range: 25–60 years). Twenty-nine HF patients were consecutively enrolled in this study. The study approval was granted by the ethics review board of the Azienda Ospedaliero-Universitaria, Arcispedale Sant'Anna, Ferrara, and informed consents were obtained in accordance with the Declaration of Helsinki of 1975. The main demographic and clinical parameters of patients were abstracted from clinical records and are reported in Supplementary Table 1 (see Supplementary Material available online at http://dx.doi.org/10.1155/2014/257081). HF diagnosis was based on history of HF of at least six months duration, reduced exercise tolerance, objective left ventricular functional impairment (LVEF), and raised level of N-terminal pro-Brain natriuretic peptide (NTpro-BNP) above the normal range at hospital entry. Diagnosis of idiopathic dilated cardiomyopathy was based on accepted criteria [11]. HF staging was performed by the New York Heart Association (NYHA) classification and on the basis of NTpro-BNP value. Patients were receiving standard evidence-based guided pharmacological treatment [11].

Serum of healthy individuals and of HF patients was obtained from blood samples collected from an antecubital vein. After clot formation, samples were centrifuged at 3000 rpm for 15 min and serum was immediately stored frozen at −80°C in single-use aliquots.

### 2.2. Isolation, Characterization, and Culture of Endothelial Cells

Human umbilical vein endothelial cells (HUVEC) were isolated from umbilical cords as previously described, with some modification [12–14]. Briefly, after cannulation and rinsing with cord buffer (PBS supplemented with 0.011 M glucose), the umbilical vein was infused with type 1 collagenase solution (0.4 mg/mL; Worthington, Lakewood, NJ) and the umbilical cord was placed for 20 min at 37°C for enzymatic digestion. Vein was flushed with warm EGM-2 medium (Lonza, Walkersville, MD) and the resulting endothelial cell suspension was centrifuged for 10 min at 150 ×g. Primary cultures of HUVECs were seeded into 25 cm^2^ flasks precoated with fibronectin (BD Bioscience, Becton Dickinson, San Josè, CA) at 5 *μ*g/cm^2^ and cultured in EGM-2 medium at 37°C in a humidified atmosphere with 5% CO_2_. The medium was changed after 24 hours and every 2 days thereafter until confluence. Primary cultures of HUVEC were dissociated with 0.025% trypsin/0.025% EDTA (Gibco BRL, Grand Island, NY) and collected by centrifugation for cell banking and/or to perform* in vitro* experiments. Cell viability was monitored by light microscopic analysis of the cell monolayers after hematoxylin-eosin staining or by quantitative examination after detachment of the monolayers by means of trypan blue dye exclusion, as described in [15, 16]. For this study, 80% of confluent HUVEC monolayers (passages 2–5) were used.

The purity of primary HUVEC cultures was evaluated by flow cytometry analysis performed on a BD FACSAria II (BD), as previously described [14, 17]. Briefly, detached cells were resuspended in 200 *μ*L of PBS containing 1% BSA (Sigma-Aldrich, Saint Luis, MO) and incubated 30 min at 4°C with the following antibodies (Ab): FITC-conjugated anti-CD146 (Miltenyi Biotec, Gladbach, Germany, Clone 541-10B2), Horizon V450-A-conjugated anti-CD144 (BD, Clone 55-7H1), PE-A-conjugated anti-CD31 (Miltenyi Biotec, Clone AC128), Alexa Fluor 647-A-conjugated anti-CD105 (BD, Clone 266), PE-Cy7-A-conjugated anti-CD34 (BD, Clone 581), APC-H7-A-conjugated anti-CD45 (BD, Clone 2D1), and Horizon v500-A-conjugated anti-CD14 (BD, Clone M5E2). Data files were collected and analysed using the FACSDiva software program (version 6.1.3; BD). Only cell suspensions characterized by endothelial cell purity equal to or greater than 98% and low levels of apoptosis, evaluated by Annexin-V/PI double staining [18, 19], were used for the* in vitro* assays.

### 2.3. Proliferation Analyses

Cell proliferation experiments were performed using the xCELLigence Real-Time Cell Analyzer (RTCA-DP version; Roche Diagnostics, Mannheim, Germany), which monitors continuously the cellular events recording label-free changes in electrical impedance (reported as cell index). Briefly, the background impedance was performed using the standard protocol provided in the software with 100 *μ*L EGM-2 complete medium (supplemented with 2% of fetal bovine serum and specific endothelial growth factors) per well, in 16-well plates. In each assay, four thousand endothelial cells (HUVEC) were seeded in 100 *μ*L of complete EGM-2 in quadruplicate in fibronectin-precoated wells and left to equilibrate at room temperature for 30 min before data recording. Cultures were grown at 37°C in a humidified atmosphere with 5% CO_2_ over-night until preestablished cell index. Then cultures were washed two times with fresh RPMI medium before the addition of RPMI medium supplemented only with 20% human serum derived from the control subjects or from the HF patients. In selected experiments, human serum was added to the cell cultures after 30 min of preincubation with neutralizing Ab anti-human VEGF (PeproTech Inc., Rocky Hill, NJ) or with control Ig (Sigma). In parallel, as control, endothelial cell cultures were exposed to recombinant VEGF_165_ (PeproTech).

Cell index of the endothelial cultures was monitored up to 66 hours. Data were analysed using the xCELLigence software (version 1.2.1) and expressed as mean ± SD of cell index normalized to the last cell index recorded before the time of cells treatment (addition of human serum).

### 2.4. Analysis of Cytokines and Chemokines

Serum samples were frozen and thawed only once before performing the MILLIPLEX MAP Human Cytokine/Chemokine Panel (Merck Millipore, Billerica, MA), a bead-based multiplex immunoassay, which allows the simultaneous detection and quantification of the following 29 human cytokines/chemokines: EGF, Eotaxin, G-CSF, GM-CSF, IFN-*α*2, IFN-*γ*, IL-10, IL-12(p40), IL-12(p70), IL-13, IL-15, 1L-17*α*, IL-1 receptor antagonist (ra), IL-1*α*, IL-1*β*, IL-2, IL-3, IL-4, IL-5, IL-6, IL-7, IL-8, IP-10 (CXCL10), MCP-1, MIP-1*α*, MIP-1*β*, TNF-*α*, TNF-*β*, and VEGF. Samples were processed following the manufacturer's recommended protocol and analyzed by using a MAGPIX instrument provided with the MILLIPLEX-Analyst Software that uses a five-parameter nonlinear regression formula to calculate cytokine/chemokine concentrations from the standard curves.

### 2.5. Statistical Analysis

Descriptive statistics were calculated. For each set of experiments, values were reported as median and/or means ± SD. The results were evaluated by using Student's *t*- and the Mann-Whitney rank-sum tests, when appropriate. Spearman's correlation coefficient was calculated to identify data correlation. Statistical significance was defined as *P* < 0.05. All statistical analyses were performed with SPSS Statistic 20 software (SPSS Inc., Chicago, IL).

## 3. Results

### 3.1. Sera from Older Healthy Subjects Promote Higher* In Vitro* Endothelial Cell Proliferation with respect to Sera from Younger Healthy Subjects

**Figure 1:**
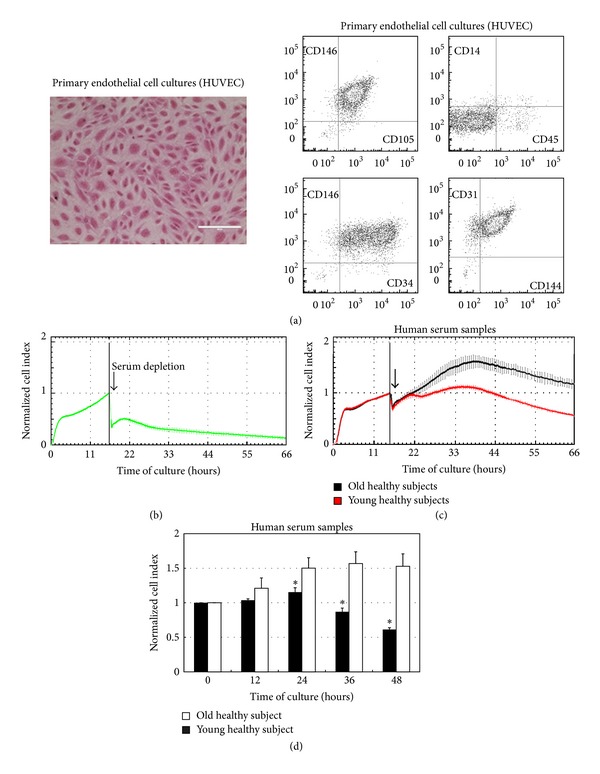
*In vitro* endothelial cell proliferation in response to serum samples from healthy subjects. Primary HUVEC cultures, characterized by multiparametric flow cytometry analysis and morphological assessment (a), were used as target for* in vitro* proliferation assays performed by dynamic monitoring of cell proliferation using the xCELLigence system, in response to human serum samples (b)–(d). In (a), a representative field of a HUVEC monolayer observed by light microscopy is shown together with a representative multiparametric flow cytometry panel, displayed as dot plots, documenting the purity of the endothelial culture. In (b), a representative profile of HUVEC proliferation in the presence of EGM-2 complete medium and after removal of growth factors and serum (arrow) is shown. In (c), two representative profiles of endothelial cell proliferation in response to serum (20%) of an old healthy donor and a young healthy subject are shown. In (b) and (c), endothelial cell proliferation is shown as cell index (mean ± SD of samples assayed in quadruplicate) after normalization to the last cell index recorded before either depletion of medium serum and growth factors (in (b), arrow) or addition of human serum samples (in (c), arrow). In (d), HUVEC proliferation in response to human serum samples obtained from old and young healthy subjects was monitored up to 48 hours and expressed as normalized cell index (mean ± SD) calculated at the indicated time points. *P < 0.05 compared to old healthy subjects at the corresponding time point.

One of the purposes of this study was to set up a reliable and reproducible biological assay using HUVEC as cellular target for monitoring the overall presence of circulating cytokines with a proangiogenic activity. For this purpose, purity of endothelial cell cultures was determined by flow cytometry analysis as cells expressing (≥98%) CD146, CD144, CD31, CD105, and CD34 and negative for CD45 and CD14 (Figure 1(a)). In order to monitor and reproducibly measure endothelial cell proliferation, we adopted a system which measures the impedance of the cell monolayer (cell index, CI) in real-time and in label-free manner without disturbing/altering the culture (Figures 1(b) and 1(c)). For this assay, endothelial cells were seeded and let to adhere and proliferate in the presence of complete medium (supplemented with 2% of fetal bovine serum and specific endothelial growth factors) until reaching a preestablished cell index value, settled in the range from 2.5 to 3.5. Subsequently, to assess and comparatively measure the effects of human serum samples on endothelial cell proliferation, medium was removed and cultures were extensively washed before the addition of medium supplemented only with 20% human serum (Figures 1(b) and 1(c)) collected from healthy subjects and HF patients. Cell index was recorded for up to 48 hours after treatment and analysis was carried out after normalization (Figures 1(b)–1(d)). In the first group of experiments, we analysed the ability of sera obtained from healthy subjects in promoting/affecting endothelial cell proliferation. In this analysis, we observed that, at the time points examined, sera obtained from older (mean age: 52 ± 7.6) subjects induced a significantly (*P* < 0.05) higher HUVEC proliferation compared to sera obtained from younger (mean age: 29 ± 8.6) healthy subjects (Figures 1(c) and 1(d)).

**Figure 2:**
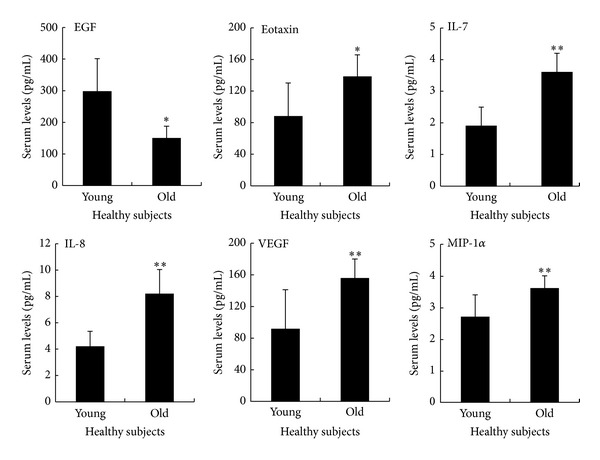
Comparative analysis of circulating cytokines/chemokines between sera obtained from young and old healthy subjects. The circulating levels of a panel of cytokines/chemokines were analyzed by multiplex immunoassay on sera obtained from healthy subjects. Data are reported as means ± SD. *P < 0.05 and **P < 0.001 compared to young healthy subjects.

This unexpected finding suggested differences between the sera of young and older healthy individuals in terms of angiogenic and/or angiostatic cytokine levels. Therefore, to check this hypothesis, we next evaluated the levels of several circulating cytokines and chemokines using the Luminex technology that allows the simultaneous detection and quantification of a panel of 29 analytes (Table 1). Sera obtained from older individuals showed significantly (*P* < 0.05) higher levels of EOTAXIN and IFN-*α*2 together with significantly (*P* < 0.001) higher levels of IL-7, IL-8, MIP-1*α*, and VEGF (Table 1 and Figure 2). On the other hand, sera from younger healthy individuals showed significantly (*P* < 0.05) higher levels only of EGF as compared to older individuals (Table 1 and Figure 2).

### 3.2. Sera of HF Patients Can Be Clustered in Two Groups on the Basis of Their Ability to Promote* In Vitro* Endothelial Cell Proliferation

We next analysed whether the HUVEC proliferation assay might also be useful to stratify sera of HF patients in relationship to relevant clinical parameters. For the purpose of this pilot study, we have enrolled 29 HF patients whose main characteristics, including cardiac functionality parameters and cardiovascular risk factors and therapy, are reported in Supplementary Table 1. Twenty-three patients (79.3%) had ischaemic aetiology, whereas 6 (20.7%) satisfied the criteria for idiopathic dilated cardiomyopathy or had HF because of hypertension and valvular disorders. All patients were receiving guidelines pharmacological therapy consisting of ACE inhibitors (55.2%), angiotensin II receptors blockers (34.5%), *β*-blockers (82.8%), antialdosterone drugs (41.4%), and diuretics (89.7%). We did not observe significant differences between NYHA class groups as for HF aetiology and the most common cardiovascular risk factors: age, diabetes, hypercholesterolemia, smoking habits, history of hypertension, and coronary diseases familiarity.

**Figure 3:**
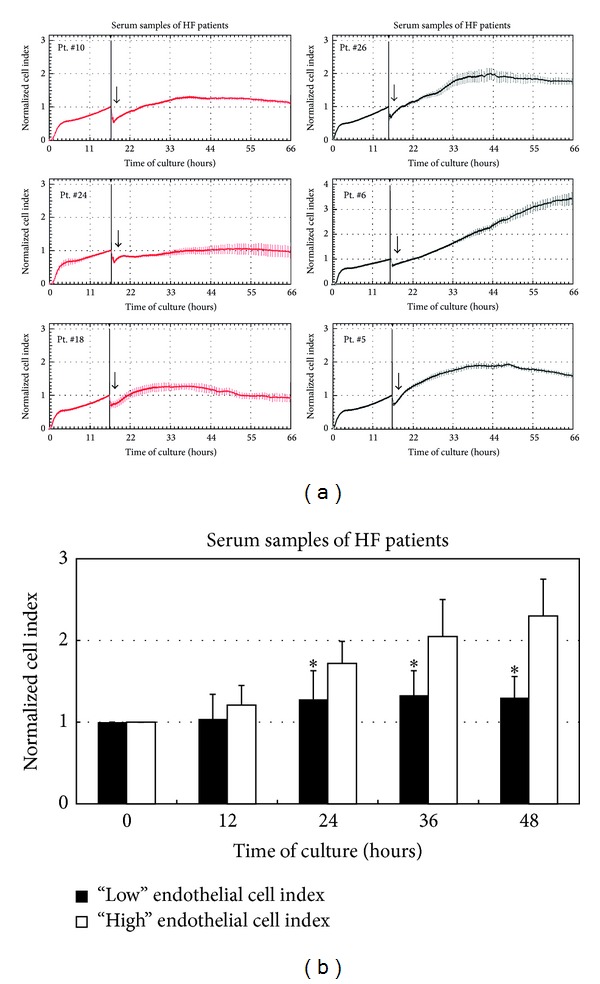
Sera of HF patients clustered on the basis of their ability to promote* in vitro* endothelial cell proliferation. HUVEC cultures were analysed for* in vitro* proliferation in response to HF patients' serum samples. In (a), representative panels of endothelial cell proliferation profiles obtained upon exposure to serum samples from HF patients (Pt.) are shown. Results are reported as normalized cell index (mean ± SD of samples assayed in quadruplicate) and show the different profiles that allowed the identification of the two distinct groups of HF patients with “high” (exemplified by Pt. number: 26, 6, and 5) and “low” (exemplified by Pt. number: 10, 24, and 18) proliferation induction ability. In (b), endothelial cell proliferation in response to HF patients' serum samples was monitored up to 48 hours and expressed as normalized cell index (mean ± SD) calculated at the indicated time points. Results are reported as mean ± SD. *P < 0.05 compared to “high” endothelial cell index group at the corresponding time point.

Based on the normalized cell index values, determined as previously described for sera from healthy subjects, we observed that sera obtained from the HF patients exhibited different effects on HUVEC proliferation, as exemplified in Figure 3(a). The results of this assay allowed us to subdivide HF samples into two groups (Figure 3(b)): a group (*n* = 18) with a high proliferation index (referred to as “high” endothelial cell index) and another group (*n* = 11) with a significantly lower proliferation index (referred to as “low” endothelial cell index). Of note, the observed differences in the ability to promote endothelial cell proliferation between the 2 HF patient subgroups were not due to differences of ages (age of the “high CI” HF patients: 69.8 ± 12.2; age of the “low CI” HF patients: 73.1 ± 8.7).

**Figure 4:**
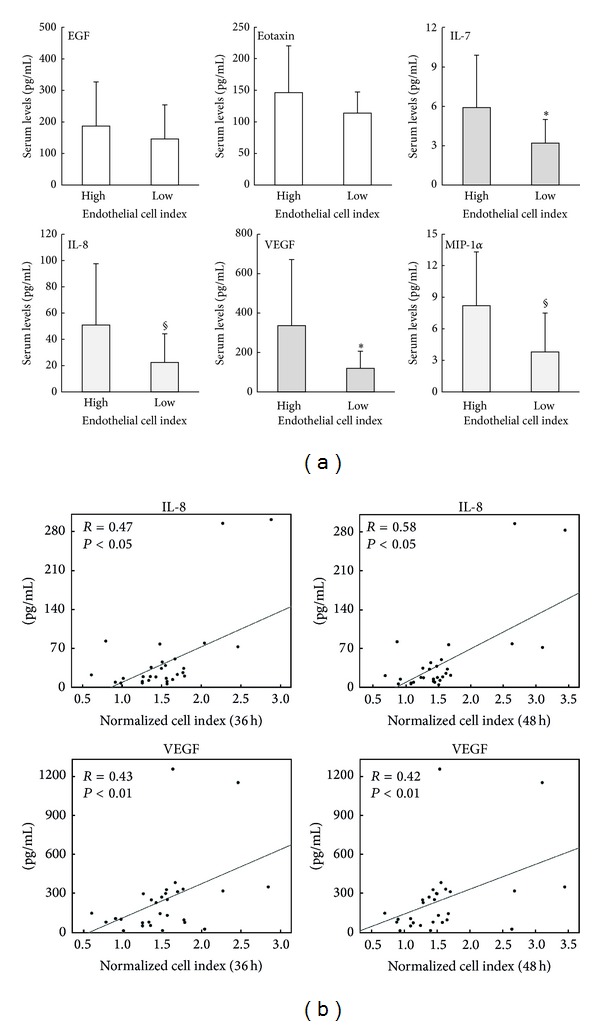
Analysis of circulating cytokines/chemokines in sera of HF patients in relationship to the* in vitro* endothelial cell proliferation response. Cytokines/chemokines levels were measured in sera of HF patients by multiplex immunoassay. In (a), the levels of the indicated cytokines measured in HF patients clustered in “high” and “low” (based on the* in vitro* endothelial cell index) are expressed as means ± SD. *P < 0.05 and ^§^ *P* < 0.06. In (b), correlation between the normalized cell proliferation index are recorded at 36 and 48 hours and the levels of IL-8 (upper panels) and VEGF (lower panels) are evaluated in the HF patients' serum samples. Correlation coefficients (*R*), calculated by Spearman's analysis, and *P* values are reported for each correlation.

In parallel, we have measured the levels of the same panel of cytokines/chemokines previously analysed in the sera of healthy subjects. As shown in Table 2, sera from HF patients belonging to the “high CI” endothelial proliferation group showed higher levels of several cytokines/chemokines with respect to patients' samples of the “low CI” endothelial proliferation group, including the angiogenic cytokines VEGF (*P* < 0.05), IL-8 and MIP-1*α* (Figure 4(a)). It is noteworthy that the levels of VEGF observed in the group “low” of HF patients (Table 2 and Figure 4(a)) were comparable to those previously observed in younger healthy donors (Table 1 and Figure 2). The important, but not exclusive, contribution of VEGF to the* in vitro* endothelial cell proliferation in response to HF patients' sera was underscored by experiments carried out using neutralizing Ab anti-VEGF (Supplementary Figure 1).

Of interest, within the cytokines/chemokines analyzed in HF patients, the levels of IL-12p70, IL-8, MCP-1, MIP-1*α*, and VEGF correlated (*P* < 0.05) positively with the endothelial proliferation index assessed in two distinct time points (36 and 48 hours), with IL-8 and VEGF showing a higher correlation (Figure 4(b)). The evaluation of potential correlation with key clinical parameters revealed no significant correlations between these cytokines and the left ventricular ejection fraction, while a significant correlation was observed between IL-12p70 and NTpro-BNP levels (*R* = 0.25, *P* < 0.05).

Comparison of the cytokine levels between the whole HF population and healthy individuals showed significantly (*P* < 0.05) higher levels of few cytokines, including IL-12p70 and IL-8, in the HF patients (Supplementary Table 2). Although the VEGF levels in the HF patients' sera were higher than the levels measured in healthy controls (254.9 ± 287.1 pg/mL versus 155.4 ± 24.6 pg/mL, resp.) the difference was not statistically significant. Anyhow, it has to be underlined that the small numbers of subjects limited the overall statistical analysis.

### 3.3. A Low* In Vitro* Endothelial Proliferation Index Is Associated with a Less Aggressive Clinical Outcome

**Figure 5:**
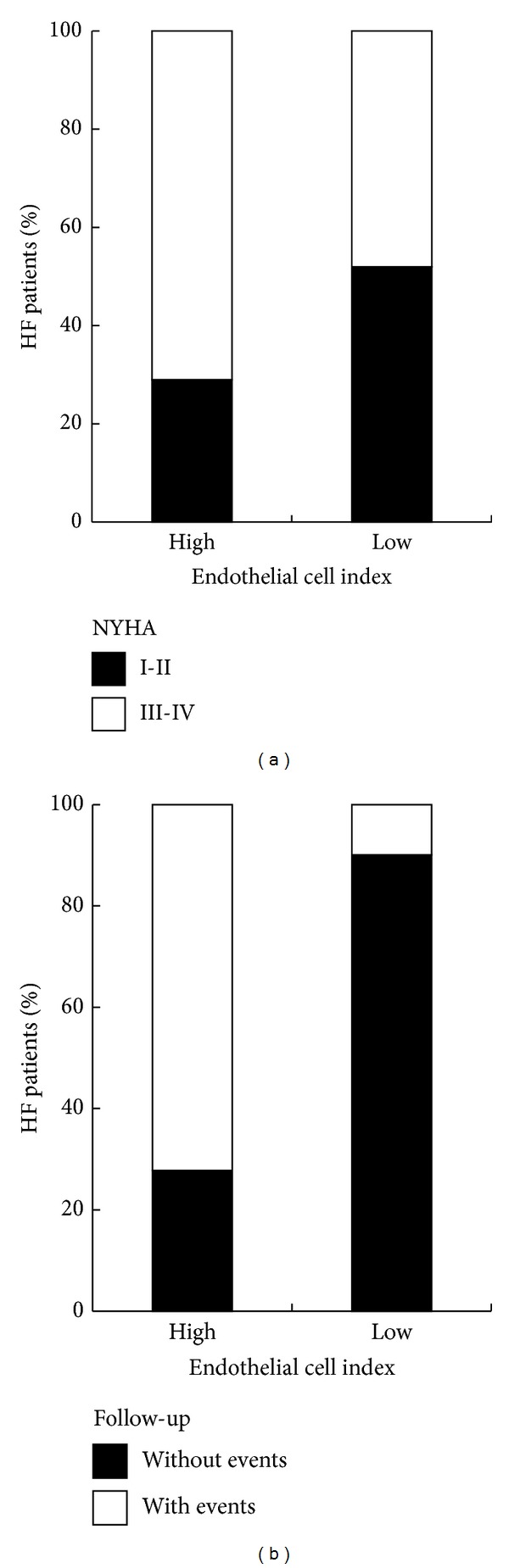
Analysis of* in vitro* endothelial cell proliferation in relationship to clinical parameters. In (a), distribution of NYHA classes (I-II and III-IV) within the high/low endothelial cell proliferation clusters of HF patients. In (b), occurrence of clinical events experienced by HF patients in two years of follow-up within the high/low endothelial cell proliferation clusters. In (a) and (b), data are expressed as percentage of HF patients.

In order to understand the potential clinical relevance of our* in vitro* endothelial proliferation assay, pointing to a subdivision of the HF patients into two groups (“high” versus “low” endothelial cell proliferation index), we first analysed the distribution of NYHA classes within the two groups. As shown in Figure 5(a), although we did not observe a significant correspondence between the classification of the HF patients in high/low and the NYHA classes, the “low” patients group was represented by a higher percentage of patients belonging to classes I-II (52% in “low CI” versus 29% in “high CI”). Moreover, the “low CI” patients showed lower levels of NTpro-BNP (median: 510.1 pg/mL; mean ± SD: 687.6 ± 357.6 pg/mL) as compared to “high CI” patients (median: 1141.5 pg/mL; mean ± SD: 1402.5 ± 743.1 pg/mL), although the difference did not reach statistical significance (*P* = 0.07). On the other hand, the two groups were very similar for the left ventricular ejection fraction (“low CI” patients: median 33%, mean ± SD 31.2% ± 7; “high CI” patients: median 34%, mean ± SD 32.7% ± 8.2, *P* = 0.3).

Next, we analysed the subdivision of the HF patients into the two groups, in relation to the clinical events they experienced in two years of follow-up. For this purpose, we considered as outcome the occurrence of major cardiovascular events: mortality (of cardiac origin), acute myocardial infarction, percutaneous transluminal coronary angioplasty, implant of defibrillator, and surgery of aortic aneurysm. Of interest, we observed a marked different distribution of the event-free HF patients between the “high CI” and “low CI” groups. In particular, while the HF patients that did not experience any major clinical cardiac events constitute the 90.1% of the group “low CI,” the majority (72.2%) of the group “high CI” was constituted by patients that experienced major events in the follow-up (Figure 5(b)).

Therefore, from these observations, it emerges a trend indicating that sera from patients with severe pathology are prone to enhance endothelial cell proliferation and, in particular, serum exhibiting high endothelial proliferation activity could indicate a higher risk for the HF patient of worsening the disease.

## 4. Discussion

In our study, by employing a standardized* in vitro* endothelial proliferation assay, we have demonstrated for the first time the following: (i) endothelial cell proliferation in response to serum samples from healthy individuals increased with age and was coupled to different serum levels of proangiogenic cytokines, including VEGF and IL-8; (ii) the endothelial cell proliferation index determined in response to serum samples from HF patients was correlated with circulating levels of several proinflammatory/proangiogenic cytokines (IL-12p70, MCP-1, MIP1*α*, IL-8, and VEGF), with IL-12p70 showing a positive correlation also with the levels of levels of NTpro-BNP; (iii) HF patient sera exhibiting high endothelial proliferation activity could indicate a higher risk for the patient of worsening the disease.

Our current findings are unprecedented and somewhat counter intuitive if one considers the important role of cardiac angiogenesis in counteracting HF. Indeed, dysfunctional blood vessel formation is a major problem in advanced HF, regardless of the aetiology [7]. However, clinical trials of VEGF gene therapy in patients with coronary artery disease or peripheral artery disease have not, to date, demonstrated clinical benefit [7]. In this respect, only few studies have tried to evaluate the levels of proangiogenic cytokines in cardiovascular patients, and mostly were carried out in patients with acute myocardial infarction (AMI) [20–26]. After an initial attempt to evaluate the predictive role of serum cytokines in patients presenting at the emergency room with chest pain and patients with AMI [22–24], Korybalska et al. [25] demonstrated that serum VEGF measured in patients with AMI (*n* = 106) was significantly higher in patients than in healthy controls and correlated with clinical and angiographic parameters. Moreover, Iribarren et al. [26] demonstrated that median serum concentration of VEGF was significantly higher (260 pg/mL) in a large cohort of patients with AMI (*n* = 695) with respect to age-matched healthy controls. Thus, although in our pilot study we have analysed only 29 HF patients, our data are in line with these previous studies obtained in patients with AMI, extending the notion that elevated levels of circulating proangiogenic cytokines are predictive of a poor prognosis, not only in patients with AMI but also in patients with established HF. In this respect, it is of interest that other studies have clearly demonstrated that elevated levels of proangiogenic cytokines, and in particular of VEGF, have a well-established pathological clinical significance in different clinical settings, such as in patients affected by different types of cancer [27, 28]. Moreover, although the role of VEGF in vascular diseases, such as atherogenesis, still remains controversial [29, 30], it has been recently demonstrated that VEGF-A gene transfer induced proatherogenic changes in lipoprotein profiles in a Apo^−/−^ mouse model [31]. Finally, the levels of circulating VEGF have also been associated to adhesion and inflammation markers in normal healthy population [32].

It is noteworthy that the* in vitro* endothelial proliferation assay, we have applied on sera derived from both healthy subjects and HF patients, represents a reproducible and reliable tool able to summarize the overall biological effects on the endothelium driven by the cytokines/chemokines milieu present in the peripheral blood. Although we are aware that the endothelial cell proliferation assessed in our assay could be the result of the contribution of additional cytokines beside those measured by our multiplex assay, our findings confirm the key role of the circulating VEGF in the promotion of endothelial cell proliferation and also suggest the potential contribution of other circulating cytokines, including the IL-12p70 [33]. It is of interest the correlation observed between IL-12p70 and NTpro-BNP that has not been previously reported in HF patients and that could deserve further investigation. Moreover, although it will be necessary to assess the* in vitro* endothelial proliferation assay in a higher number of clinical cases, our pilot study on the HF patients' sera suggests its potential prognostic value.

## Supplementary Material

Main demographic and clinical parameters of the patients included in the study.

## Figures and Tables

**Table 1:** Circulating levels of cytokines/chemokines in sera from healthy subjects.

Cytokines/chemokines	Limit of detection	Healthy subjects		*P *
		Young	Old	
EGF	3.21	236 (297.6 ± 104)	142 (149.4 ± 38.6)	<0.05
EOTAXIN	3.2	79.5 (87.9 ± 42.3)	136 (138.1 ± 27.8)	<0.05
G-CSF	3.24	22 (24.7 ± 14.3)	28.9 (30.2 ± 12.1)	n.s.
GM-CSF	1.58	3.2 (4.1 ± 1.6)	4 (3.9 ± 0.8)	n.s.
IFN-*α*2	0.72	3.8 (4 ± 1.8)	5.1 (5.7 ± 0.7)	<0.05
IFN-*γ*	1.37	1.8 (6 ± 9.1)	4.2 (4.6 ± 0.4)	n.s.
IL-10	0.89	<OOR	<OOR	/
IL-12p40	0.36	<OOR	<OOR	/
IL-12p70	1.6	<OOR	<OOR	/
IL-13	0.82	<OOR	<OOR	/
IL-15	1.06	<OOR	<OOR	/
IL-17*α*	1.28	<OOR	<OOR	
IL1ra	0.36	4.8 (6.7 ± 5.3)	4.2 (5 ± 0.8)	n.s.
IL-1*α*	0.87	<OOR	<OOR	/
IL-1*β*	0.81	<OOR	<OOR	/
IL-2	1.44	<OOR	<OOR	/
IL-3	/	<OOR	<OOR	/
IL-4	1.17	<OOR	<OOR	/
IL-5	1.04	<OOR	<OOR	/
IL-6	0.63	20.2 (20.2 ± 27.4)	<OOR	n.s.
IL-7	0.62	1.8 (1.9 ± 0.6)	3.3 (3.6 ± 0.6)	<0.001
IL-8	1.51	3.7 (4.3 ± 1.2)	8.1 (8.4 ± 1.9)	<0.001
IP-10	3.21	197 (231.7 ± 96.3)	173 (172.9 ± 39.9)	n.s.
MCP-1	3.21	436 (448.7 ± 124.4)	573 (574.8 ± 56.9)	n.s.
MIP-1*α*	0.9	2.7 (2.7 ± 0.7)	3.7 (3.6 ± 0.4)	<0.001
MIP-1*β*	3.21	30.2 (37.3 ± 12)	34.1 (35.5 ± 9.4)	n.s.
TNF-*α*	1.26	5.03 (5.17 ± 1.9)	5.4 (5.9 ± 1.7)	n.s.
TNF-*β*	1.31	<OOR	<OOR	/
VEGF	3.2	59.8 (91.3 ± 49.8)	155 (155.4 ± 24.6)	<0.001

Values are expressed as pg/mL and reported as median (mean ± SD). OOR<: out of (below) detection range; n.s: nonstatistically significant; *P* < 0.05: statistically significant.

**Table 2:** Circulating levels of cytokines/chemokines in sera from HF patients stratified based on the endothelial cell proliferation index (“high CI” versus “low CI”).

Cytokines/chemokines	HF patients		*P*
	“High CI”	“Low CI”	
EGF	126.5 (186.9 ± 140)	104 (145.9 ± 108.4)	n.s
EOTAXIN	141.5 (146.3 ± 74)	117 (113.8 ± 33.6)	n.s
G-CSF	25.4 (43.3 ± 45.9)	23.1 (26.5 ± 16.5)	n.s
GM-CSF	5.5 (6.1 ± 2.9)	3.7 (4.7 ± 2.7)	n.s
IFN-*α*2	6.7 (7.4 ± 4.9)	2.6 (4.5 ± 4.9)	n.s
IFN-*γ*	3.3 (4.9 ± 5.5)	1.5 (1.6 ± 1.7)	<0.05
IL-10	0.4 (4.9 ± 8.6)	0.2 (4.3 ± 9.6)	n.s
IL-12p40	<OOR	<OOR	/
IL-12p70	2.1 (4.2 ± 6.5)	0.2 (2.5 ± 4.3)	n.s
IL-13	<OOR	<OOR	/
IL-15	1.5 (2.6 ± 4.7)	0.3 (2.9 ± 5.6)	n.s
IL-17*α*	<OOR	<OOR	/
IL1ra	11.3 (25.1 ± 26.2)	6.5 (8.7 ± 9.3)	n.s
IL-1*α*	1 (6.3 ± 15.8)	1.5 (7.4 ± 15.6)	n.s
IL-1*β*	<OOR	<OOR	/
IL-2	<OOR	<OOR	/
IL-3	<OOR	<OOR	/
IL-4	<OOR	<OOR	/
IL-5	<OOR	<OOR	/
IL-6	5.7 (7.5 ± 7.3)	1.6 (12.8 ± 17.3)	n.s
IL-7	4.7 (5.9 ± 4)	2.9 (3.2 ± 1.8)	<0.05
IL-8	34.1 (50.9 ± 46.7)	16.3 (22.4 ± 21.8)	<0.06
IP-10	293 (620.9 ± 610)	342 (357.4 ± 127.9)	n.s
MCP-1	663.5 (697 ± 448)	471 (485 ± 155)	n.s
MIP-1*α*	4.5 (8.2 ± 5.1)	3.8 (3.8 ± 3.7)	<0.06
MIP-1*β*	37.3 (39.9 ± 22.6)	36.5 (52 ± 48.2)	n.s
TNF-*α*	10.9 (12.8 ± 7.5)	8.7 (9.3 ± 3.3)	n.s
TNF-*β*	<OOR	<OOR	/
VEGF	290 (336.1 ± 335.5)	85.6 (119.6 ± 86.3)	<0.05

CI: cell index. Values are expressed as pg/mL and reported as median (mean ± SD). OOR<: out of (below) detection range; n.s: nonstatistically significant; *P* < 0.05: statistically significant; *P* < 0.06: close to statistically significant.
