# Supplementary material for: In Vitro Endothelial Cell Proliferation Assay Reveals Distinct Levels of Proangiogenic Cytokines Characterizing Sera of Healthy Subjects and of Patients with Heart Failure
Source: Mediators Inflamm. 2014 Mar 23;2014:257081. doi: 10.1155/2014/257081 (PMC3981563; doi:10.1155/2014/257081)
Supplement: Supplementary file 1 — Main demographic and clinical parameters of the patients included in the study. [file 257081.f1.pdf]

Supplementary Table 1. Main demographic and clinical characteristics of the HF patients (n=29).

Variables	Values*
NYHA I-II/III-IV (n)	12/17
Age (years)	72.2±9.6
Male/Female (n)	24/5
BMI (kg/m^2)	26.7±4.7
Heart rate (bpm)	74.7±18.9
Ischemic aetiology (%)	79.3
EDD (mm)	61.8±7.9
EDV (mL)	209±68.3
LVEF (%)	32.2±7.7
NTpro-BNP (pg/ml)	134.5±151.0
Risk factors (%):	
Diabetes	27.6
Hypercholesterolaemia	55.2
Smoking habits	65.5
History of hypertension	91.3
CAD familiarity	31
Therapy (%):	
ACE inhibitors	55.2
ARB	34.5
B-blockers	82.8
Anti-aldosterone	41.4
Diuretics	89.7
Digitalis	3.4
Nitrates	31
Calcium antagonists	6.9

*Values are expressed as either number or means±SD, or percentage (%) as indicated.

NYHA: New York Heart Association; BMI: Body Mass Index; EDD: End-Diastolic Diameter; EDV: End-Diastolic Volume; LVEF: Left Ventricule Ejection Fraction; CAD: Coronary Artery Disease; ACE: Angiotensin-Converting Enzyme; ARB: Angiotensin II-Receptor Blockers.
